# Editorial: Synergistic advances in gene technology, nanobiotechnology, and photonic innovations for next-generation diagnostics and therapeutics

**DOI:** 10.3389/fbioe.2025.1685142

**Published:** 2025-08-28

**Authors:** Zhi Chen

**Affiliations:** Department of Chemistry, Korea University, Seoul, Republic of Korea

**Keywords:** gene technology, nanobiotechnology, photonic innovations, precision diagnostics, therapeutic nanomaterials

## Introduction

The past two decades have seen extraordinary advances in our ability to interrogate, manipulate, and treat biological systems at the molecular level. Gene technology, nanobiotechnology, and photonic innovations—once largely separate domains—are now converging into a unified framework that is redefining both diagnostics and therapeutics. This convergence is driven by shared goals: to detect disease earlier, to act with higher precision, and to adapt interventions in real time. As global health challenges continue to evolve, from emerging infectious diseases to cancer heterogeneity, the integration of these disciplines offers a pathway toward truly personalized, predictive, and preventive medicine.

## From gene-level precision to real-time sensing

Gene technology has progressed well beyond the foundational discovery of CRISPR-Cas systems. Today, genome engineering tools are not only used for permanent genetic modification but are also being harnessed for diagnostic purposes, capable of detecting specific nucleic acid sequences with single-base resolution. CRISPR-based biosensing platforms—particularly when integrated with optical or electrochemical readouts—can achieve rapid, label-free detection of genetic disorders, viral mutations, and minimal residual disease. This paradigm shifts diagnostics from centralized laboratory settings toward portable, decentralized, and even at-home applications, empowering earlier and more frequent monitoring.

## Nanobiotechnology as the functional bridge

While genetic tools provide the specificity, nanobiotechnology contributes the versatility and multifunctionality needed for real-world deployment. Advances in the synthesis of two-dimensional materials, quantum dots, and self-assembled nanostructures have enabled exquisitely tunable optical, electrical, and biochemical properties. These materials can act as transducers, amplifying biological recognition events into measurable signals, or as therapeutic agents, delivering payloads with spatial and temporal precision. The capacity to design nanomaterials with responsiveness to multiple stimuli—pH, temperature, light, or enzymatic activity—creates opportunities for theranostic systems, where detection and treatment are seamlessly linked in a single platform.

## Photonic innovations: from imaging to intervention

Photonics has long been central to biomedical imaging and spectroscopy, but recent developments are extending its reach into therapeutic domains. High-speed modulators, compact laser systems, and integrated photonic circuits are enabling real-time processing of biosignals, multiplexed detection, and adaptive feedback control. In therapy, photonic energy sources—from visible to near-infrared—are being exploited to trigger targeted drug release, induce photothermal or photodynamic effects, and even modulate neural or genetic activity through optogenetics. The intersection of photonics with gene technology and nanobiotechnology is particularly fertile, as it allows light to serve as both a diagnostic probe and a therapeutic trigger.

## Addressing the remaining bottlenecks

Despite these advances, several bottlenecks remain. Sensitivity and specificity in complex biological matrices are still limited by background noise and off-target interactions. Scalability and reproducibility in nanomaterial synthesis remain challenging, particularly for regulatory approval and clinical translation. Photonic systems, while increasingly compact, must balance power, penetration depth, and safety for *in vivo* use. Integration of these components into a single, user-friendly device requires overcoming not only technical barriers but also logistical and regulatory hurdles, especially in resource-limited settings.

## This research topic: a reflection of the field’s momentum

The five contributions gathered in this Research Topic provide a representative cross-section of the interdisciplinary advances driving the integration of gene technology, nanobiotechnology, and photonic innovations. Each embodies a different facet of the overarching goal: to combine molecular specificity, functional nanostructures, and precision optical control into next-generation diagnostic and therapeutic platforms.

One direction represented in this Research Topic is the rational design of nanomaterials with tunable photonic properties for biomedical intervention (*Regulating the Size of Antimony Nanoparticles to Enhance the Photoresponse in the Near-infrared Region and Anti-hepatoma Cell Activity*) Huang et al. The work on size-regulated antimony nanoparticles exemplifies how controlling morphology at the nanoscale can precisely adjust localized surface plasmon resonance to match therapeutic laser wavelengths, thereby enhancing both photothermal and photodynamic effects. By demonstrating that semimetallic nanomaterials—traditionally underexplored in biomedicine—can deliver high photothermal conversion efficiencies while maintaining biocompatibility, this study aligns perfectly with the Research Topic’s emphasis on material-driven advances in light-based therapeutics.

Another contribution highlights the enabling role of advanced photonic devices in biomedical contexts (*High-Speed Electro-Optic Modulator with Group Velocity Matching on Silicon Substrate*) Liu et al. The development of a high-speed thin-film lithium niobate electro-optic modulator, achieving over 110 GHz bandwidth with low driving voltage, illustrates how device architectures originally conceived for telecommunications can be adapted to meet the demands of real-time biosignal processing. Such capabilities are increasingly relevant for high-throughput genetic analysis, rapid pathogen detection, and multiplexed biosensing, where the capacity to handle large volumes of optical data without latency can fundamentally change diagnostic workflows.

In the area of nanobiotechnology and therapeutic delivery, the Research Topic includes work elucidating the molecular mechanisms underlying the therapeutic action of complex natural products (*Investigating the molecular mechanisms of the “Astragalus-Codonopsis” herb pair in treating diabetes: a network pharmacology and bioinformatics approach with molecular docking validation*) Yang et al. The study on the Astragalus–Codonopsis herb pair applied a multi-tiered approach—combining network pharmacology, molecular docking, and dynamics simulations—to identify GSK3β as a critical target in modulating the insulin receptor signaling pathway. This integrative methodology bridges traditional medicine and modern computational biology, offering insights that could inform both small-molecule drug design and biomolecular engineering.

Complementing this mechanistic perspective, two contributions focus on advanced delivery platforms designed to overcome the limitations of conventional formulations. One study reports the creation of pH-responsive supramolecular hydrogels from asiaticoside, whose nanofibrous architecture enhances transdermal flux and skin retention of hydrophobic therapeutics. This demonstrates how natural molecules can be repurposed as self-assembling scaffolds, integrating responsive behavior and sustained release into a single system (*Supramolecular nanofibers of natural asiaticoside for self-supporting gelation and enhanced transdermal delivery*) Hu et al. The other presents a nanoemulsion gel incorporating Sophora alopecuroides oil, engineered for improved skin permeability and potent anti-biofilm activity against both *S. aureus* and MRSA. By tackling microbial resistance while optimizing drug delivery, this work illustrates how nanoscale formulation strategies can address multifactorial therapeutic challenges (*Nanotechnology-Driven Nanoemulsion Gel for Enhanced Transdermal Delivery of Sophora alopecuroides L. Empyreumatic Oil: Formulation Optimization, and Anti-Biofilm Efficacy*) Cheng et al.

These contributions underscore the breadth of innovation occurring at the nexus of molecular recognition, functional materials, and optical control. They collectively embody the shift toward integrated systems in which sensing, processing, and intervention are co-designed.

## Future directions: toward intelligent, adaptive platforms

Looking ahead, several trends are poised to reshape the landscape. First, the integration of artificial intelligence with biosensing and imaging will enable adaptive diagnostics that learn from each patient’s data, refining both detection thresholds and therapeutic strategies in real time. Second, advances in wearable and implantable biosensors will allow continuous molecular monitoring, providing a temporal dimension to diagnostics that is currently missing from most clinical workflows. Third, the development of smart nanomaterials that respond dynamically to disease-associated cues—releasing drugs, altering optical signatures, or modulating gene expression—will bring us closer to closed-loop therapeutic systems. Finally, regulatory frameworks will need to evolve to keep pace with these rapidly converging technologies, ensuring both safety and timely access to innovation.

The path forward will require more than incremental improvement; it will demand deep collaboration across scientific, engineering, and clinical communities. By continuing to bridge molecular specificity, nanoscale functionality, and photonic precision, we can create diagnostic and therapeutic systems that are not only powerful but also accessible, adaptable, and truly transformative for patient care. The works gathered here are a testament to the ingenuity driving this transformation and a preview of what is yet to come.

